# Scrutinizing Virus Genome Termini by High-Throughput Sequencing

**DOI:** 10.1371/journal.pone.0085806

**Published:** 2014-01-20

**Authors:** Shasha Li, Hang Fan, Xiaoping An, Huahao Fan, Huanhuan Jiang, Yubao Chen, Yigang Tong

**Affiliations:** 1 State Key Laboratory of Pathogen and Biosecurity, Beijing Institute of Microbiology and Epidemiology, Beijing, China; 2 Beijing Computing Center, Beijing, China; Plymouth University, United Kingdom

## Abstract

Analysis of genomic terminal sequences has been a major step in studies on viral DNA replication and packaging mechanisms. However, traditional methods to study genome termini are challenging due to the time-consuming protocols and their inefficiency where critical details are lost easily. Recent advances in next generation sequencing (NGS) have enabled it to be a powerful tool to study genome termini. In this study, using NGS we sequenced one iridovirus genome and twenty phage genomes and confirmed for the first time that the high frequency sequences (HFSs) found in the NGS reads are indeed the terminal sequences of viral genomes. Further, we established a criterion to distinguish the type of termini and the viral packaging mode. We also obtained additional terminal details such as terminal repeats, multi-termini, asymmetric termini. With this approach, we were able to simultaneously detect details of the genome termini as well as obtain the complete sequence of bacteriophage genomes. Theoretically, this application can be further extended to analyze larger and more complicated genomes of plant and animal viruses. This study proposed a novel and efficient method for research on viral replication, packaging, terminase activity, transcription regulation, and metabolism of the host cell.

## Introduction

Tailed bacteriophages use a specific mechanism on their tails during attachment to the host bacterial surface receptor to perform recognition, adsorption, adhesion and baseplate positioning procedures [1]. After adsorption onto the host cell, the bacteriophage delivers its genomic DNA into the host cell through its tail channels [2]. In the lytic mode, genomes of some phages are circularized through complementary protrusions in the termini and the standard bacterial “theta” mode is employed for circular-DNA replication [1]. Some circular DNA subsequently adopt the rolling-circle replication mechanism to generate a number of head-to-tail DNA concatemers which serve as substrates for viral DNA packaging [2].

During bacteriophage packaging, the concatemeric DNA is cleaved by terminase, and then encapsulated into a preformed icosahedron protein shell called “prohead”. In most dsDNA bacteriophages and viruses such as herpes viruses, poxviruses and adenoviruses [2], the general packaging process includes recognition of a specific packaging site on the concatemeric DNA by the packaging enzyme (*pac* for T4,SPP1, and P22; *cos* for T7, T3, and λ) followed by a cut at or near the site to initiate the packaging process. After translocating one unit length of genome DNA into the prohead using ATPase activity of the packaging enzyme, the concatemeric DNA is once again cut to generate the other terminus, which terminates the packaging process. The packaging enzyme consists of a small and a large subunit [2], [3] and since it generates both the termini, it was named “terminase”. The small subunit recognizes the concatemeric DNA and recruits itself to the large subunit for cleavage initiation. The large subunit has a prohead-binding activity, which docks the prohead’s portal vertex, an ATPase activity that translocates the cleaved DNA into the protein shell, and a nuclease activity, which cuts the concatemeric DNA and generates the genome terminus [4].

Because of their specific nuclease activities, terminases from different bacteriophages create different types of terminal sequences [5]. Based on the genomic termini, at least eight types of dsDNA bacteriophages and viruses have been classified. These include: i) lambdoid phages with 5′ protruding cohesive ends, ii) bacteriophages Φ105, HK97, and D3 with 3′ protruding cohesive ends, iii) bacteriophages T7, T3, Ye03-12$, and A1122 with direct terminal repeats and no circular permutation, iv) headful packaging phages SPP1, P22, and P1 with both terminal redundancy and circular permutation, v) bacteriophages T4, ES18, and sf6 with terminal redundancy and circular permutation but no obvious *pac* site, vi) bacteriophage Φ29 family and adenoviruses with direct terminal repeats and protein adhering to each end of the genomic DNA, vii) Mu-like and B3 phages with host DNA fragments at each end of the phage genome molecule, and viii) N4-like phages with short and variable length direct terminal repeats with a unique sequence at the left genome termini and several different sequences at the right genome termini [5], [6], [7], [8].

Phage and viral genomes have many types of termini, but only two packaging mechanisms have been identified thus far: *cos* mode and headful mode. *cos* refers to the packaging of one genome length DNA and headful indicates the packaging of 102%–110% of the genome DNA [2]. The genomic DNA of phage lambda, T7, T3, and herpes viruses package in *cos* mode where the terminase introduces staggered nicks at the *cosN* site to generate cohesive ends. This initiates the packaging process, followed by recognition and cut at another *cosN* site, which terminates the first packaging process and initiates another packaging process [5]. In this mode, the cleavage sites are specific and the packaged genomic DNA lengths are accurate [3], [5]. The genomic DNA of phages, like P22, SPP1, P1, and T1, is packaged in the headful mode where the terminase recognizes and cleaves at a specific *pac* site to generate the first termini to be packaged into the prohead. After the prohead is fully filled with a headful of DNA, a sequence-independent cleavage is carried out on the concatemeric DNA at a random site downstream of the another *pac* site to complete the packaging process [9], [10]. Thus the headful mode does not always have sequence specificity [3], [8], [9], [10], [11], [12]. It is long believed that in T4-like bacteriophages, there is no specific *cos* or *pac* site, and that the genome is generated by headful mode where cleavage occurs at random sites to produce both phage genome termini [11], [12].

High-throughput or next generation sequencing (NGS) techniques have developed rapidly in the last few years and are widely used in various fields of biomedical research. Recently, we sequenced the genome of T4-like bacteriophage IME08 with NGS and reported the occurrence of some sequences at unexpected high frequencies. These sequences had some interesting characteristics, leading us to hypothesize that they were terminal sequences of the phage IME08 genome, and that they may have been generated by sequence-specific cleavage of the pre-genomic DNA (concatemeric DNA, which could be cut by the terminase into genomic DNA) [13]. In this study, we used NGS to sequence adaptor-linked genomic DNA of T3 and T4-like phages IME09 to test our hypothesis and used this technique to study other dsDNA phages. In total, we sequenced one iridovirus genome and twenty phage genomes, and our work revealed that the high frequency sequences (HFSs) found in the NGS reads are indeed the terminal sequences of viral genomes, and that the ends of the iridovirus and T4-like phage genomes have preferred sequences rather than random sequences, which is unique and different from previous findings [14]. Evidences are also provided to demonstrate that other bacteriophages have either specific or random terminal sequences. Further, we established a criterion to distinguish the type of termini and the viral packaging mode in these bacteriophages, and detected additional details of terminal sequences such as terminal repeats, secondary termini, and multi-termini. Using these techniques, we determined for the first time the presence of a unique left end and random right ends in *S. aureus* phages, and show that the same terminase cleavage sites and terminal structure existed in this kind of phages. Thus, this study showed that the NGS technique can simultaneously determine the complete sequence of viral genomes as well as their terminal sequences, and that this technique can serve as a practical tool to study viral replication and packaging mechanisms.

## Materials and Methods

### Phages and Bacterial Strains

**Figure 1:**
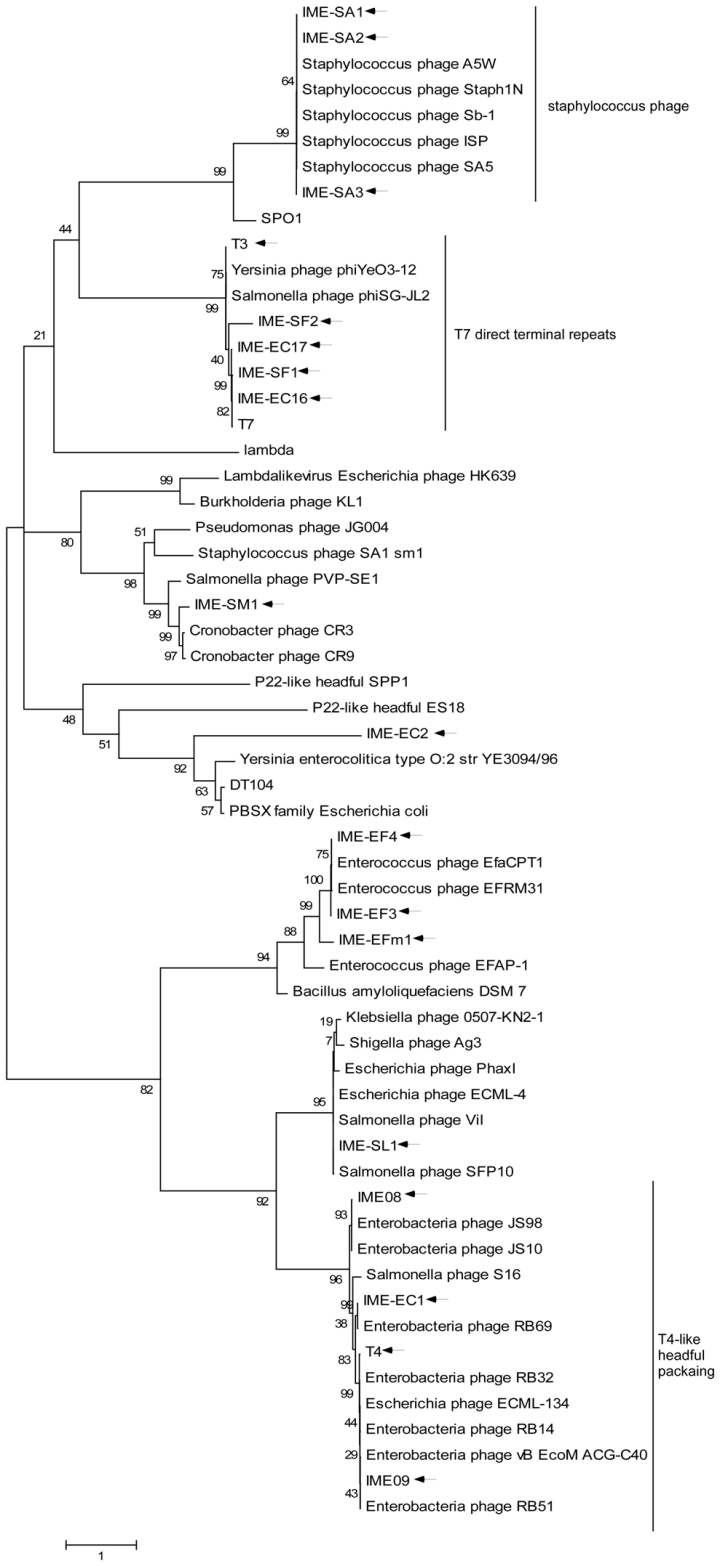
Phylogenetic analysis of large terminase subunits from 17 phages sequenced in this study and their comparison to other phages with known packaging mechanisms. Phages sequenced in this study are indicated by arrows. The tree was generated from an amino acid alignment (gap open cost, 10; gap extension cost, 1; end gap cost, free) using the Maximum Likelihood method with 1000 bootstrap replicates (MEGA 5.10).

The lytic bacteriophage strains IME08, IME09 and IME11 were isolated from sewage collected from a local hospital (PLA Hospital 307, Beijing, China), and the T3 bacteriophage (ATCC 11303-B3) was obtained from ATCC (http://www.ATCC.org). Iridovirus, W150 was isolated from infected mosquitos (collected from Yunnan province, China, in 2012) and cultured in *Aedes albopictus* C6/36 cells. The host bacteria for IME08 and IME09 was *Escherichia coli* strain 8099 which is a disinfection reference strain widely used in China [15]. For the IME11 bacteriophage, an *E. coli* strain isolated from a patient in PLA Hospital 307 was used as the host. Other phages, such as IME-EC-16, IME-EC-17, IME-EC1, IME-EC2, IME-AB2, IME-AB3, IME-SA1, IME-SA2, IME-SA3, IME-EF3, IME-EF4, IME-EFm1, IME-SF1, IME-SF2, IME-SM1, IME-SL1 were all lytic bacteriophages isolated from PLA Hospital 307 sewage and the host bacteria for these bacteriophages were isolated from clinical samples in PLA Hospital 307. Table 1 shows a list of these viruses and their respective host bacteria. Figure 1 shows the genus of relative phages. The accession numbers of the phage genomes are: T3 - KC960671, IME08 - NC_014260, IME09 - NC_019503, IME11 - NC_019423, IME-AB2 - JX976549. No specific permits were required for the described field studies because of the collaboration of the PLA hospital 307 and our laboratory; the samples collected were not privately owned or protected and did not involve endangered or protected species.

**Table 1:** Viruses subjected to high-throughput sequencing.

Phage	Family	Genus	Genome length (bp)	Host bacteria
T3	Podoviridae	T7-like	38,208	*Escherichia coli* K12
IME08	Myoviridae	T4-like	172,253	*Escherichia coli* 8099
IME09	Myoviridae	T4-like	166,499	*Escherichia coli* 8099
IME11	Podoviridae	N4-like	72,570	*Escherichia coli*
IME-EC-17	Podoviridae	T7-like	38,435	*Escherichia coli*
IME-EC-16	Podoviridae	T7-like	38,870	*Escherichia coli*
IME-EC1	Myoviridae	T4-like	170,335	*Escherichia coli*
IME-EC2	NA	NA	41,510	*Escherichia coli*
IME-AB2	Myoviridae	NA	43,665	*Acinetobacter baumannii*
IME-AB3	Myoviridae	NA	43,050	*Acinetobacter baumannii*
IME-SA1	Myoviridae	Twortlikevirus	140,181	*Staphyloccocus aureus*
IME-SA2	Myoviridae	Twortlikevirus	140,434	*Staphyloccocus aureus*
IME-SA3	Myoviridae	Twortlikevirus	140,807	*Staphyloccocus aureus*
IME-EF3	Siphoviridae	NA	41,118	*Enterococcus faecalis*
IME-EF4	Siphoviridae	NA	40,685	*Enterococcus faecalis*
IME-EFm1	NA	NA	42,598	*Enterococcus faecium*
IME-SF1	Podoviridae	T7-like	38,842	*Shigella flexneri*
IME-SF2	Podoviridae	T7-like	40,387	*Shigella flexneri*
IME-SM1	NA	NA	149,960	*Serratia marcescens*
IME-SL1	Myoviridae	Viunalikevirus	153,667	*Salmonella*
Iridovirus W150	Iridoviridae	Iridovirus	162,590	Aedes albopictus C6/36 cells

### Isolation of Bacteriophages

Bacteriophages were isolated from sewage using enrichment cultures [16]. All phages used in this study were isolated using the same method. For example, to isolate IME-SA1, approximately 2 mL of filtered (Millipore membranes, pore diameter 0.45 µm) sewage was mixed with 2 mL of 3×LB and 100 µl overnight culture of *S. aureus* (isolated from clinical samples in the same local hospital as the sewage). The enrichment culture was incubated at 37°C for at least 14 hours with agitation to allow amplification of bacteriophages, and was then centrifuged (10 min, 10,000 g, 4°C). The supernatant was filtered (Millipore membranes, pore diameter 0.45 µm) to remove the residual bacterial cells and 200 µl of bacteriophage was mixed with 500 µl of *S. aureus* cells in the exponential growth phase (OD_600_ = 0.2 to 0.5) and incubated at 37°C for 30 min. About 5 mL of top agar (LB with 0.7% agar) at 50°C was then added, and the mixture was poured onto an LB plate prewarmed at 37°C (double-layer method) [16]. The plates were then incubated at 37°C overnight to obtain bacteriophage plaques [17].

### Genomic DNA Extraction from Bacteriophages

Bacteriophage DNA was extracted based on the method described by Sambrook, et al [18]. In brief, 250 µl purified phage particles were added to a polypropylene centrifuge tube and treated with DNase I (1 µg/ml) (Takara Bio, Shiga, Japan) and RNase A (1 µg/ml) (Takara Bio, Shiga, Japan) for 30 minutes at room temperature, and then incubated at 80°C for 15 min to inactivate the nucleases. EDTA (pH 8.0, 20 mM), proteinase K (50 µg/ml) and SDS (0.5%) were added to the digestion mixture followed by incubation for 1 hour at 56°C. The solution was then deproteinated twice by extraction with an equal volume of phenol-chloroform-isoamyl alcohol (25∶24∶1). The protein-free DNA in the aqueous phase was precipitated with an equal volume of isopropanol (AR grade) by incubation overnight at –20°C, followed by centrifugation at 12,000 *g* (10 min, 4°C). The precipitated DNA was washed twice with 75% ethanol, and finally dissolved in 100 µl deionized water.

### Ligation of Adaptor Sequences to the Bacteriophage Genomic DNA Ends

To determine the terminal sequences of the bacteriophage genome, we designed a pair of complementary oligonucleotides (Forward: 5′-AGTGTAGTAGT -3′. Reverse: 5′-CTACTACACT-3′) with the reverse primer phosphorylated at the 5′ end (Sangon Biotech, Shanghai, China). The two oligonucleotides annealed together, forming a double stranded adaptor with a ‘T’ overhang at the 3′ terminus. The termini of the bacteriophage genome were polished blunt with T4 DNA polymerase (IonTorrent, San Francisco, USA), phosphorylated at the 5′ end with T4 Polynucleotide Kinase (IonTorrent) and a base ‘A’ was added at the 3′ end with Taq DNA polymerase (IonTorrent) using standard protocols. The adaptors were then ligated to the modified termini of bacteriophages in a reaction mixture containing 25 µl end-repaired genomic DNA sample, 1 µl annealed adaptor, 1 µl T4 DNA ligase (IonTorrent) and 10 µl 10×ligase buffer (IonTorrent) followed by incubation at 25°C for 10 min, and the ligated DNA was purified using AMPure beads (Beckman Coulter, California, USA).

### High-throughput Sequencing

The genomes of T3 and IME11 were sequenced using Solexa HiSeq2000 Genome Analyzer (Illumina, San Diego, USA) at the Beijing Genomics Institute. The genomes of IME08, IME09, IME-EC-16, IME-EC-17, IME-EC1, IME-EC2, IME-AB2, IME-AB3, IME-SA1, IME-SA2, IME-SA3, IME-EF3, IME-EF4, IME-EFm1, IME-SF1, IME-SF2, IME-SM1, IME-SL1, and IME-MS1 were sequenced using the semiconductor sequencer in Life Technologies Ion Personal Genome Machine (PGM) Ion Torrent sequencer (IonTorrent). The sequencing protocol for the Illumina HiSeq2000 included: shredding the complete bacteriophage genome into small fragments, ligation of these small DNA fragments with forked adaptors followed by hybridization on the sequencing chip through the forked adaptor, and finally bridge amplification on the chip as previously described [19]. The amplification was performed by sequencing-by-synthesis method using four fluorescently labeled nucleotides that were added to the flowcell channels during DNA synthesis. The florescent light signals were detected by the Genome Analyzer, which performed basecalling [20]. The protocol for PGM sequencing was also similar to the HiSeq2000 except that instead of bridge amplification, emulsion PCR was used, where PCR is carried out in a water-in-oil microreactor containing a single DNA molecule on a bead and the H^+^ ion torrent signal is detected during sequencing-by-synthesis [21].

### Bioinformatics Analysis

Complete genome sequences of bacteriophages were assembled either with Velvet [22], ABYSS [23], SOAPdenovo [24], or CLC genomics workbench (Aarhus, Denmark). Fastx toolkit [25] (http://hannonlab.cshl.edu/fastx_toolkit/) was used to remove the adaptor sequences. Clean sequences with adaptors removed or raw sequences without adaptor tags were mapped onto the phage genome with CLC genomics workbench. Then, the occurrences of every nucleotide from the start of the reads were counted using an in-house python script. Blast search was performed on NCBI website to find each phage’s similar sequence, genome sequences with low homology or sequences that differed completely from other sequences were defined as new phages. Genome annotation were performed with RAST (Rapid Annotation using Subsystem Technology [26]). Conserved CDSs such as large terminase subunit were selected for phylogenetic analysis using MEGA 5.10 and subsequently the phages were assigned to specific genera.

## Results

### High Frequency Sequences Represent Genome Termini

**Figure 2:**
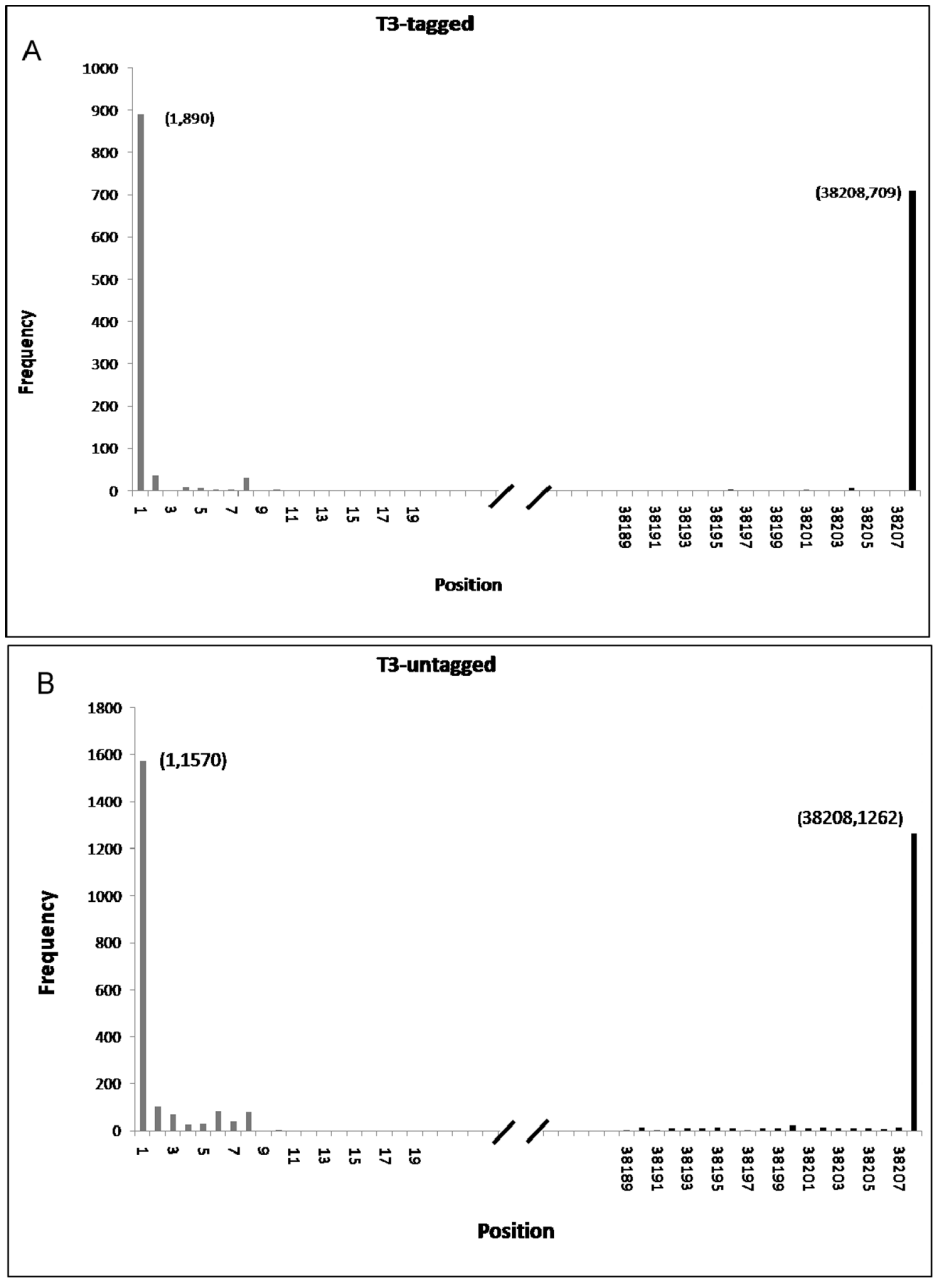
Frequencies of reads representing the genome termini of phage T3. (**A**) Adaptor tagged genomic DNA. (**B**) Un-tagged genomic DNA. Strand orientation and nucleotide numbering are adopted from the complete genome sequence record of T3 phage in GenBank (NC_003298).

**Figure 3:**
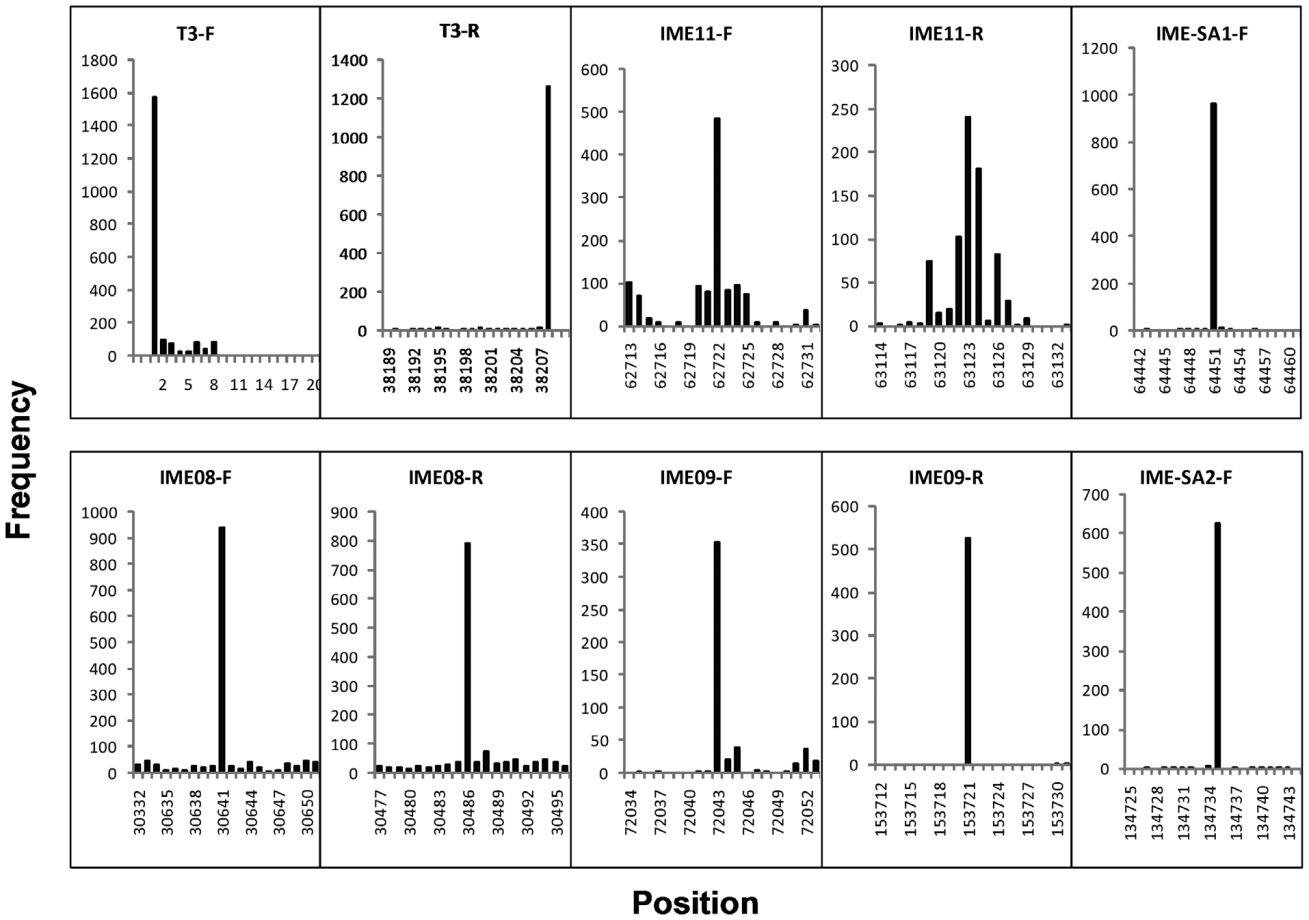
The highest frequency sequence and their surrounding 20 bp. Sequences with extraordinary high frequencies (with low frequencies of nearby sites) can be called true termini such as in T4-like phage IME08, IME09 and *Staphylococcus aureus* phage IME-SA1. N4-like phage IME11 have distinctive terminal sequences at the left while at the right end there are two termini separated by two bases.

Our previous work suggested that the high frequency sequences (HFSs) that resulted from high-throughput sequencing represent the termini of sequenced genome [13]. To further confirm this occurrence, we chose a well-studied T3 phage as a model and sequenced its genome using NGS. For this, the termini of the genomic DNA were tagged with a synthetic dsDNA adaptor and another sample of un-tagged T3 phage genome was also subjected to NGS. To determine the characteristics of the adaptor containing sequences, these sequences were first extracted from the raw sequencing dataset. After removal of the adaptors, the sequences were mapped onto the T3 phage genome and the individual frequencies of every base from the start of reads were calculated with a home-made python script. Mapping showed that the terminal sequences at both the 5′ and 3′ ends had extraordinarily high occurrences (890 at 5′ and 709 at 3′) compared with the internal sequences which had an average frequency of 1.35. Such tagging of internal sequences occurred randomly by tag ligation of broken genomic DNA (Figure 2A). For the untagged T3 genomic DNA sample, the average occurrence of the internal bases at read starts was 5.12 times, however the 5′ and 3′ terminal base occurrences were 1570 and 1262 times respectively, which were about 300 times higher than the average occurrence (Figure 2B, Table 2). The highest frequencies of the un-tagged forward and reverse sequences were identical to the adaptor-tagged highest frequencies of the forward and reverse sequences (Table 2), and matched exactly with the previously reported terminal sequences of T3 phage (NC_003298). This suggests that terminal sequences of genomes generated by NGS have higher occurrences than the internal sequences. Our results thus confirm that the high frequency sequences in high-throughput sequencing represent the terminal sequences of input genomic DNA, which is consistent with our previous study [13]. Note that in these analyses a terminus (HFS) was determined as real by comparing the height of its peak with the peaks of the surrounding 20 bp because the peak of a terminus will be evidently higher than the rest of positions on the whole genome. Note that 20 bp was chosen arbitrarily although it could be any length such as 10 bp, 500 bp. If an extraordinarily high peak was exhibited by a terminus but not by the surrounding sites, then it was determined a true terminus (Figure 3).

**Table 2:** Sequences with the highest frequencies in bacteriophage T3 genome.

Sample	Direction	Average frequency	Highest frequency	Sequence	First base position
Tagged	Forward	1.35	890	TCTCATAGTTCAAGAACCCA…	1
	Reverse	1.35	709	AGGGACACATAGAGATGTAC…	38208
Un-tagged	Forward	5.12	1570	TCTCATAGTTCAAGAACCCA…	1
	Reverse	5.12	1262	AGGGACACATAGAGATGTAC…	38208

In the complete genome of T3 phage sequenced by Illumina (KC960671), we identified 19 indels (insertion-deletions) by comparing with the T3 phage sequence available in GenBank (NC_003298, sequenced by ABI 377 DNA analyzer) (Table S1). Multiple differences were observed between our NGS data and the GenBank sequence including gene positions, and variations in nucleotide bases and amino acids. These differences are listed in the supplementary material (Table S1). These discrepancies are likely due to conventional low coverage sequencing techniques used in the previous study or by mutations that could have accumulated during propagation. Therefore, NGS technology could also be a useful tool to update sequence data generated by conventional sequencing strategies, in addition to being a quick and affordable tool to perform large scale sequencing.

### Terminal Characteristics of the N4-like IME11 Genome

**Figure 4:**
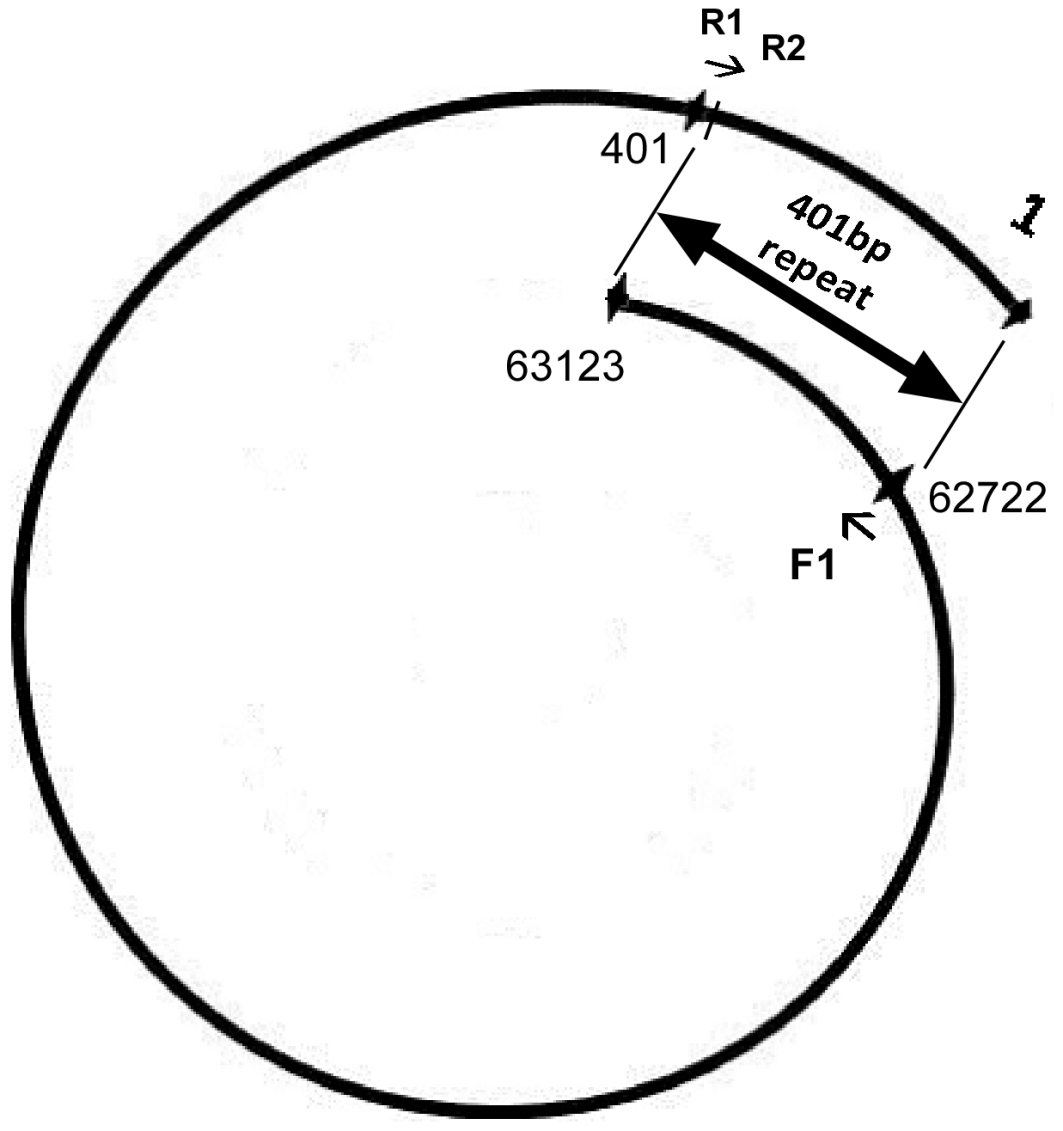
Schematic map of 401 bp direct terminal repeats in the N4-Like phage IME11 genome. F1 (62722) is the forward cleavage site in the genome (packaging from 1 to 62722). R1 (401) and R2 are the reverse cleavage sites (packaging from 63123 to 401).

**Figure 5:**
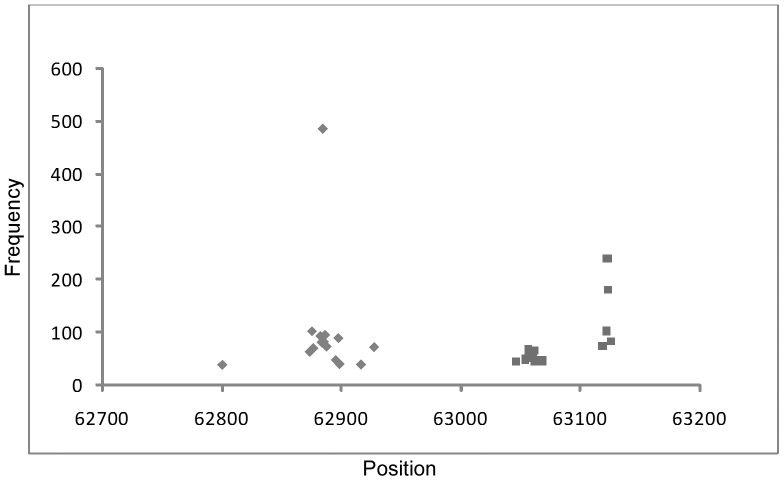
Distribution of the top 15 forward and reverse HFSs in the N4-like bacteriophage IME11 genome. One HFS is on the left end while the other two are on the right end. Their frequencies are 486, 240 and 180, respectively (black rhombus: forward, gray square: reverse).

It has been reported that the terminal sequence of N4-like bacteriophage genomic DNA is distinct compared to the genomic ends of other phages such as T4, T7, λ, Φ29, Mu-like, and SPP1 [6], [8], [27]. One of the characteristics of N4-like phage genome is the presence of short direct terminal repeats with variable lengths (390–440 bp) at the 3′ protrusions [5], [6], [28]. In this study, we isolated a N4-like *Escherichia coli* bacteriophage, IME11, and sequenced its genomic DNA by high-throughput sequencing (JX880034). The complete genome sequence was assembled and the raw sequences were mapped to the assembled genome. Occurrences of all sequences in the raw sequencing data were counted, which demonstrated the presence of high frequency sequences in both forward and reverse directions (Table 3). These forward and reverse sequences were 401 bp apart (Figure 4, Figure 5), indicating that they are terminal sequences of the genome and that there are 401 bp direct terminal repeats at the ends of the IME11 bacteriophage consistent with previous reports [5], [6]. Note that IME11 has two peaks on its reverse sequence which is likely because it is separated from the right end by only 2 bp (Table3).

**Table 3:** The top 10 forward and reverse sequences in the N4-like IME11 phage genome.

Frequency	Position	Orientation	Read sequence
486	62722	F	AATCTTTGCAGAAATCTTCG…
240	63123	R	GTGGCTGGGGCTACACCCGG…
180	63124	R	TGTGGCTGGGGCTACACCCG…
102	63122	R	TGGCTGGGGCTACACCCGGA…
101	62713	F	CGTTCTCTGAATCTTTGCAG…
94	62724	F	TCTTTGCAGAAATCTTCGAT…
92	62720	F	TGAATCTTTGCAGAAATCTT…
88	62735	F	ATCTTCGATACTTTTCTTAC…
82	63126	R	GGTGTTGGCTGGGGCTACAC…
81	62723	F	ATCTTTGCAGAAATCTTCGA…

Previous studies have demonstrated that the genomes of N4-like phages are cleaved by terminases using different mechanisms [5]. These differences in terminal processing produce asymmetric termini at the ends of the genome: the left end is a unique sequence, while at the right end six groups of terminal sequences with permutations have been identified thus far. By analyzing the high frequency sequences in IME11, a unique forward sequence was determined as the left terminus, while for the right terminus, two high frequency sequences were identified which were only two bases apart (Table 3, Figure 5). It is likely that IME11 is a novel N4-like phage with only two closely located terminal sequences at the right end of its genome and therefore different from the previously reported N4-like phages. Another possible reason could be that the overall coverage of our sequencing was not high enough and therefore resulted in the relatively low numbers of terminal sequences.

### T4-like Phage Terminase Likely Cleaves Genomic DNA in a Sequence Preferred Manner

**Figure 6:**
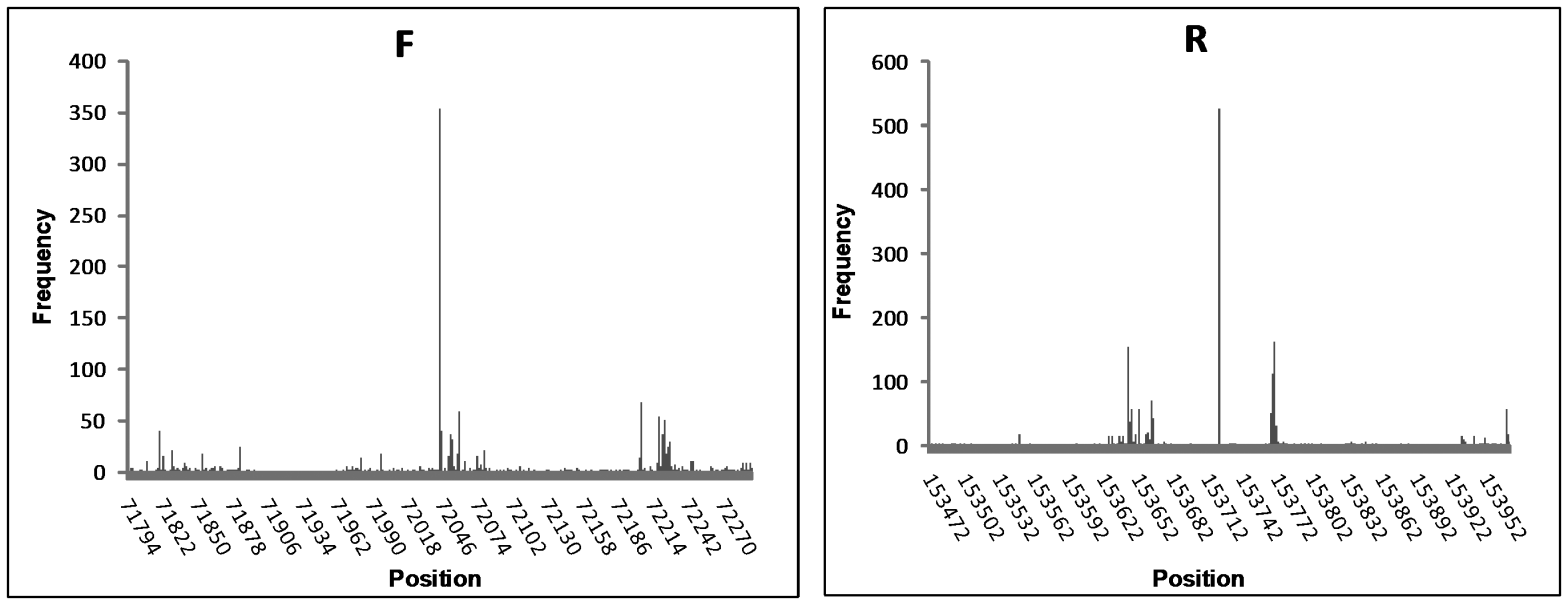
The highest frequency sequences (forward – F, reverse – R) with the surrounding 500 bp. Although most 500(F) and 527 times (R).

In a previous study that determined the genomic sequence of T4-like phage, IME08, we fortuitously identified HFSs in the raw sequencing data and hypothesized that these HFSs were not randomly generated by genome fragmentation but were the pre-existing termini of the genome [13]. In the present study, the well characterized T3 phage with pre-determined termini was sequenced by NGS as described above to confirm this hypothesis. Further, to characterize the termini of T4-like phage genome, we applied NGS to sequence the genome of IME09, a T4-like phage isolated in our laboratory. To replicate the results obtained with T3 sequencing, both the adaptor-tagged and untagged IME09 genome were subjected to NGS. As a comparison with IME08/T3/N4 which were sequenced with Illumina sequencer GAII or Hiseq2000, IME09 was sequenced with the desktop sequencing machine, Ion Torrent. The complete genome sequence of IME09 was assembled with both tagged and untagged sequences and the raw sequence reads were mapped against the assembled genome. This was followed by calculation of individual sequence occurrences in IME09. The results showed the presence of 1,239,578 reads in the tagged sequence dataset indicating that each sequence occurred at an average of 3.7 times ( = N_r_/L_g_/2. N_r_ (total reads number): 1239578; L_g_ (genome length): 166499; directions: forward and reverse) if the terminal sequences in the genome were generated randomly by the terminase. Although the average occurrence was ∼ 4 times, a number of sequences (312 and 60 sequences in the file without/with adaptor tagged respectively, Table S2) were present more than 100 times (Table 4, Figure 6). Though nearly 96% of all sequences occurred 1–9 times, the highest frequency sequence had up to 527/256 occurrence in the reads without/with adaptor tag (Table S2). Further analysis indicated that the HFSs in the file with/without adaptor contain identical sequences (Table 4). These sequences were unique since they occurred only once in the assembled genome. BLAST analysis revealed that these sequences were homologous to unique sequences from other evolutionarily related T4-like phages such as RB51, T4 (Figure 1). Moreover, the 20 bp read sequences upstream and downstream of the highest frequency sequence in the genome occurred at normal frequencies (4 times) making the HFS an extraordinarily high peak (Figure 3). In addition, no obvious repeat sequences were observed in the assembled genome of IME09. Together, these results suggest that the HFS reads were not randomly generated during library construction because if they were such artifacts, individual reads would have similar fragmentation opportunities and would eventually occur at the same frequency rather than multiple HFSs and reads peaks.

**Table 4:** The top 10 forward and reverse sequences in T4-like IME09 phage genome.

Sequence	F1^1^	F2^2^	Orientation	Position	Junction^3^
GTTACATAGCTTTTATTCTGTCCAGCACGC…	527	256	R	12779	cgctttaaca**g**ttacatagc
GTAATTATTGGACACGTGAAGAAGCAATGA…	382	239	R	7461	gaccaatata**g**taattattg
GTTTCAACTGAATAGTCTGTATGAGACTGT…	376	218	R	80630	gttttcatca**g**tttcaactg
GTTCAATTTACTGCTGCCTTGGCTTCACAG…	374	205	R	59498	tggaacccca**g**ttcaattta
GTATCATTGGATTTTTGACCAGGACGAACT…	353	186	F	72043	tgtaatatga**g**tatcattgg
GTACCATTAGTACGAAGTCCTACCACTTCA…	343	185	F	6994	aacacgacca**g**taccattag
GTTTCTCCATCGACCAGAGAACCGTCAACA…	329	173	F	106911	tgtagtagta**g**tttctccat
GTTTCAATAATCTTTCTTTCTTCTTCGGAC…	326	171	F	13694	tttacgatta**g**tttcaataa
GTTCCTGGCTGGATTGGTGAATCTGCCGTG…	320	168	F	156168	aattgtaggt**g**ttcctggct
GAAATATTAACTGATTCGAAAATACTATCA…	310	168	R	65575	tgcttccgga**g**aaatattaa

^1^ Frequency/without adaptor,

^2^ Frequency/with adaptor,

^3^ g is the start site of HFSs.

**Figure 7:**
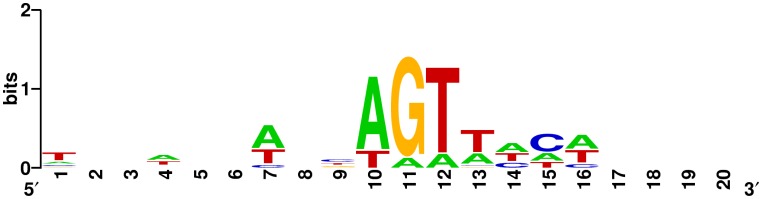
Consensus sequence of the top 10 HFSs together with their upstream sequence. Height of the base represents the degree of conservation. Start site of HFSs is at position 11.

The top 10 forward and reverse HFSs with adaptor tags were mapped on the T4-like phage, IME09 genome. In these HFSs, 20 bp together with their upstream sequences (Column 5 of Table 4) were chosen to plot the consensus sequence using Weblogo [29]. As shown in Figure 7, the first base of these top 10 HFSs is G. Further, analysis of the first base of all sequences with/without adaptor tag indicated that the first base of HFSs were dominated by G and A, and as the occurrence increased, G exceeded A as the main start base (Figure S1). In adaptor tag read sequences with 1–4 occurrences, the first base percentage was comparable with the genome base composition (Figure S1), however, A and G comprised more than 90% of the first base in sequences occurred more than 11 times, and T or C were never observed in sequences that occurred more than 100 times (Figure S1). These differences in the first base between HFSs and normal frequency sequences in part suggested the sequence preference of T4-like phage, IME09 terminase.

In addition, the consensus sequence analysis (Figure 7) showed the presence of a conserved sequence, A|GTTACA, in the top 10 HFSs, which probably represented the preferred recognition and cleavage site of terminase to start or complete a packaging process. Moreover, the HFSs of T4-like phages IME08, IME09, and IME-EC1 have distinct consensus sequences compared to phages with completely permuted circular genome such as IME-AB2 (Figure S2). However, note that the number of consensus ‘cutting sites’ on IME09 genome could not be defined accurately. The results calculated using degenerate motif finder (http://www.insilicase.co.uk/Web/DegenerateSites.aspx) indicated that 25,227 consensus sequences were found on the whole genome of phage, IME09 (166,499 bp) (Table S3). HFS reads of IME09 could not be observed in all these consensus sequences but part of them, only 19,215 sequences were matched bettween HFSs and consensus sequences in the genome of IME09 (Table S3). In conclusion, the results mentioned above possibly suggest that the cleavage of T4-like phage terminase is sequence preferred, which is not consistent with previous reports that T4-like phage terminase cleaves the genome randomly to produce random termini during packaging [2], [3], [10], [11], [12]. The same phenomenon was previously reported in T4-like phage, IME08 [13]. The likely explanation for this inconsistency may be that the terminase is only sequence-preferred, but not strictly sequence-specific (see discussion below).

### Characteristics of Phage Termini Determined by NGS

Large scale sequencing using NGS allowed us to simultaneously obtain the complete sequence of the viral genomes as well as to detect their terminal sequences. The occurrences of these terminal sequences were estimated by calculating the ratio of the highest frequencies to the average frequency of the phage sequences, i.e. R (ratio) = highest frequency/average frequency. Using this formula, we successfully distinguished the type of termini that occurs in one iridovirus and 20 bacteriophages including iridovirus W150, T3, N4-like phage IME11, *Serratia marcescens* phage IME-SM1, *Salmonella* phage IME-SL1, *Escherichia coli* phage IME-EC2, T4-like phage IME08 and IME09, IME-EC1, *Acinetobacter baumannii* phage IME-AB2, IME-AB3, *Enterococcus faecalis* phage IME-EF3, IME-EF4, *Enterococcus faecium* phage IME-EFm1, T7-like *Escherichia coli* phage IME-EC-16, IME-EC-17, T7-like *Shigella flexneri* phage IME-SF1, IME-SF2 and *Staphylococcus aureus* phage IME-SA1, IME-SA2, IME-SA3. All these phages were isolated in our laboratory and sequenced by NGS. For example, in *A. baumannii* bacteriophage IME-AB2 and IME-AB3, and *Salmonella* phage IME-SL1, R <30 indicates absence of termini suggesting a circular genome or a completely permuted and terminally redundant circular genome. In iridovirus W150, T4-like phages IME08, IME09 and IME-EC1 where 30< R <100, preferred termini exist with terminal redundancy and partially circular permutations are created by sequence preferred cleavage of terminase. Note that the reason for the low coverage in iridovirus W150 was because it was difficult to concentrate the virus as well as extract its genomic DNA. However, in T7-like phage T3, IME-EC-16, IME-EC-17, IME-SF1, IME-SF2, *E. faecalis* phage IME-EF3, IME-EF4, *S. aureus* phage IME-SA1, IME-SA2 and IME-SA3, N4-like bacteriophages IME11, *Escherichia coli* phage IME-EC2, *Enterococcus faecium* phage IME-EFm1, and *Serratia marcescens* phage IME-SM1 where R >100, fixed termini exist with terminase recognizing specific sites at the two ends that results in absence of terminal redundancy or circular permutation (Table 5). Based on these findings we propose that the type of termini that exist in various organisms can be estimated from the ratio between the highest frequencies and the average frequency of sequences obtained from NGS, particularly in organisms with smaller genomes such as bacteriophages where the termini play a critical role in infection.

**Table 5:** Summary of the criteria used to distinguish termini.

Phage	Nr^0^	Coverage^1^	G(bp)^2^	A^3^	H^4^	Ratio(H/A)	T1-F/T2-F^5^	T1-R/T2-R^6^	Characteristic	Termini^7^
IME-AB2	320,569	1194	43,665	3.67	44	11.99	1.1	1.16	no obvious termini	circular genome
IME-AB3	513,242	3466	43,050	5.96	160	26.85	1.04	2.25	no obvious termini	
IME-SL1	595,966	1106	153,667	1.94	16	8.25	1.07	1.07	no obvious termini	
IME08	9,883,185	6924	172,253	28.69	938	32.69	1.18	1.28	multi preferred termini	preferred termini
IME09	2,096,452	2355	166,499	6.30	527	83.65	1.03	1.38	multi preferred termini	
IME-EC1	1,005,020	1866	170,335	2.95	109	36.95	1.5	1.38	multi preferred termini	
Iridovirus W150	86,689	667	162,590	0.27	24	88.89	1.2	1.85	low coverage, with terminal redundancy, multi-preferred termini, 3′ end less permuted than 5′	
IME11	686,863	2479	72,570	4.73	486	102.75	4.81	1.33	left unique, right permuted with two termini, with 401 bp direct terminal repeats	fixed termini
IME-EF4	431,269	6582	40,685	5.3	2762	521.13	1.09	19.59	right unique with one terminus, left random with no obvious termini	
IME-SA2	281,925	1730	140,434	1.00	626	626.00	6.59	1.30	left unique with one terminus, right random with no obvious termini, with 8 kb LTR	
IME-SA1	1,474,251	3577	140,181	5.26	961	182.70	11.44	1.17	left unique with one terminus, right random with no obvious termini, with 8 kb LTR	
IME-SA3	54,737	285	140,807	0.19	90	473.68	12.86	1.12	left unique with one terminus, right random with no obvious termini, with 8 kb LTR	
T3	574,358	5606	38,208	7.52	1570	208.78	15.39	10.02	unique termini at both ends, with 230 bp direct terminal repeats	
IME-EC-16	509,962	7106	38,870	6.56	2342	357	35.48	38.38	unique termini at both ends, with 147 bp direct terminal repeats	
IME-EC-17	311,780	3040	38,435	4.06	897	220.94	15.74	4.85	unique termini at both ends, with 81 bp direct terminal repeats	
IME-EC2	785,489	7259	41,510	9.46	1075	106.02	9.43	1.02	a definite start site with a few bases variation and partially permuted ending sites with a terminal redundancy of about 300 bp	
IME-EF3	367,251	3638	41,118	4.66	1125	241.42	30.83	4.95	unique termini at both ends, with 540 bp direct terminal repeats	
IME-EFm1	1,103,508	9657	42,598	12.95	3855	297.68	19.67	14.23	unique termini at both ends, with 265 bp direct terminal repeats	
IME-SF1	355,397	4675	38,842	4.57	1609	352.1	67.04	32.49	unique termini at both ends, with 159 bp direct terminal repeats	
IME-SF2	720,811	9155	40,387	8.92	1934	216.82	9.57	9.08	unique termini at both ends, with 190 bp direct terminal repeats	
IME-SM1	162,104	1081	149,960	0.54	420	777.78	42	7.76	unique termini at both ends, with 487 bp direct terminal repeats	

^0^ Number of reads,

^1^ Maximum coverage,

^2^ Genome-length,

^3^ Average frequency ( = Nr/G/2),

^4^ Highest frequency,

^5^ Forward top1 frequency/Forward top2 frequency,

^6^ Reverse top1 frequency/Reverse top2 frequency,

^7^ Termini type. Criterion: R<30 (no termini, circular genome), 30<R<100(preferred termini), R>100 (fixed termini). T1/T2>3(unique termini), T1/T2<3(multi-termini, or no termini).

**Figure 8:**
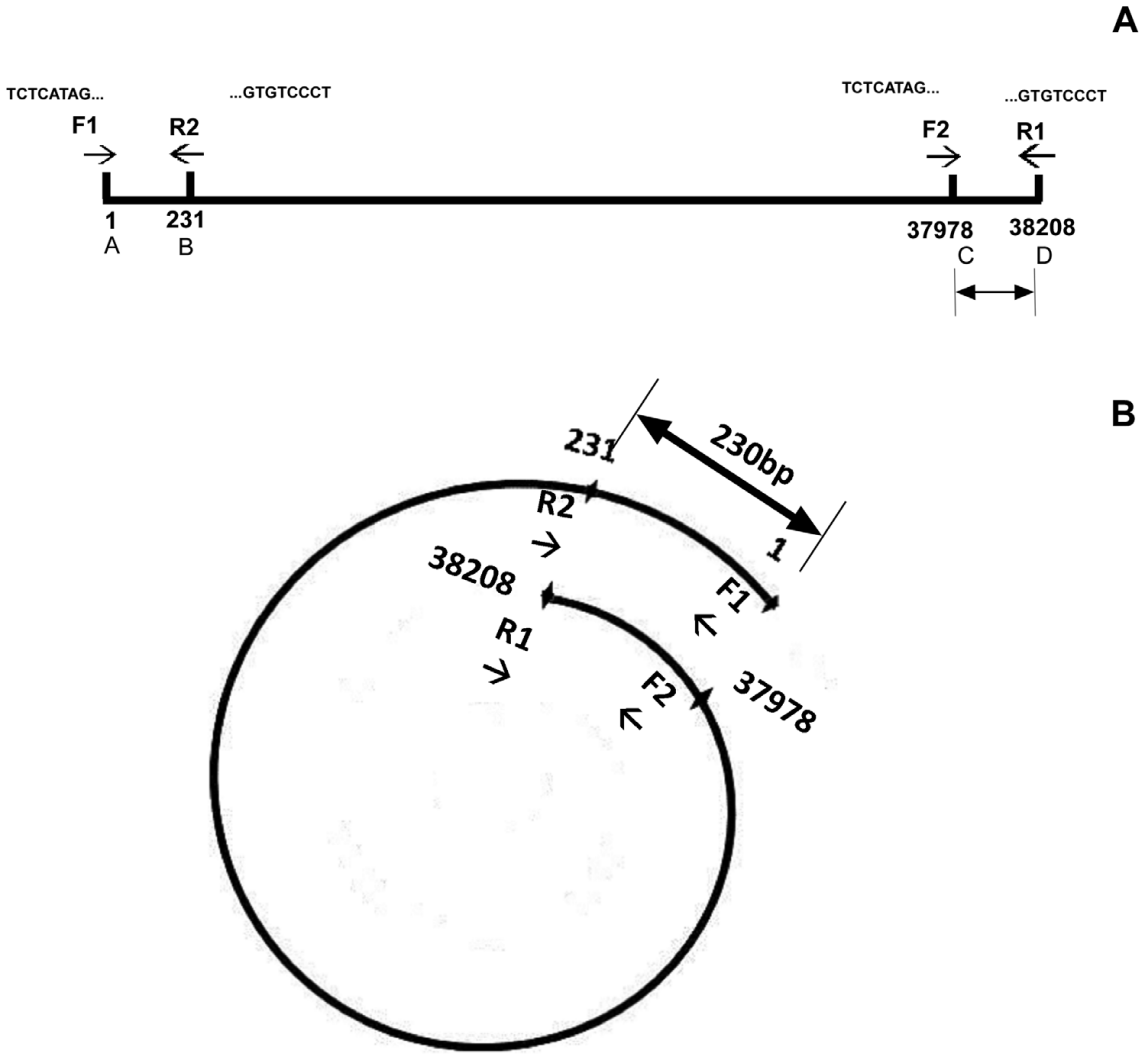
Schematic map of direct terminal repeats and forward/reverse terminase cleavage sites distribution in bacteriophage T3. (A) The whole genome length of bacteriophage T3 is 38,208 bp. There are two forward and reverse terminase cleavage sites at 137,978 and 23,138,208 respectively, which indicates that there are 230 bp repeats on the T3 genome, both A – C or A – D can serve as the whole genome. (B) The same as (A), both F1/R1 and F2/R2 can serve as the forward and reverse cleavage sites. There are 230 bp repeats on bacteriophage T3 genome. F1/F2: TCTCATAGTTCAAGAACCCA, R1/R2: AGGGACACATAGAGATGTAC.

**Figure 9:**
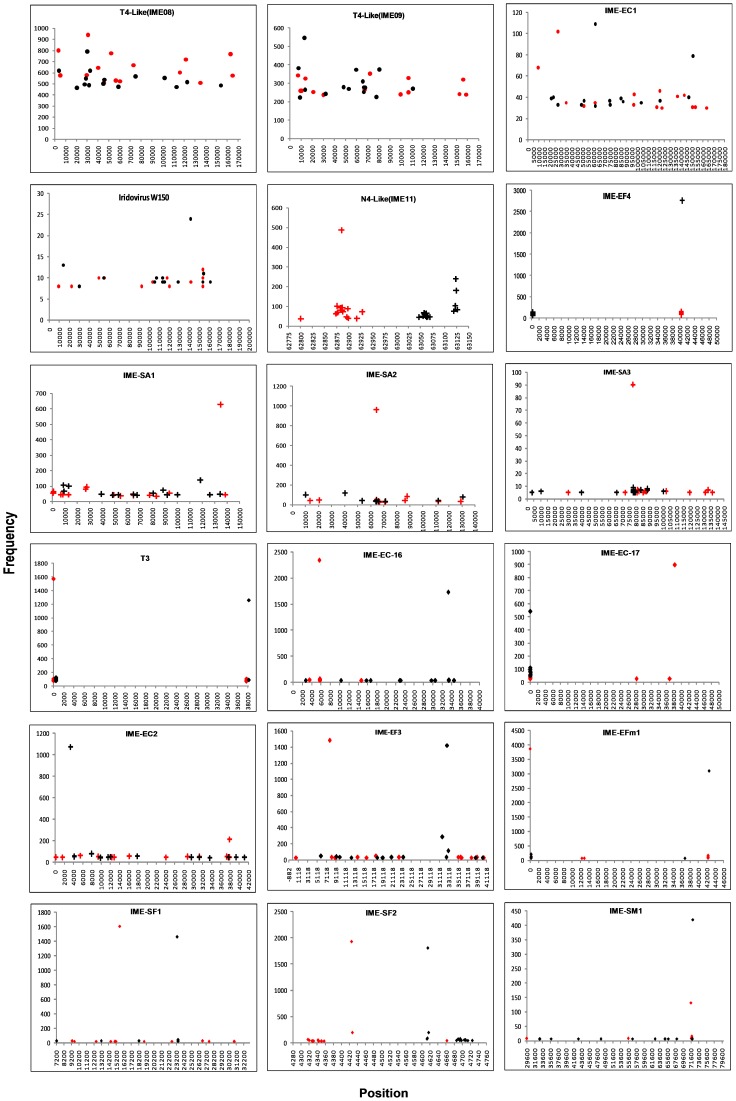
The top 15 forward and reverse HFSs of indicated phage genomes. T4-like phages IME08, IME09, IME-EC1, Iridovirus W150 have preferred termini, which have multi-termini throughout the entire genome. T7-like phage T3, IME-EC-16, IME-EC-17, IME-SF1, IME-SF2, *E. faecalis* phage IME-EF3,IME-EF4, *S. aureus* phage IME-SA1,IME-SA2 and IME-SA3, N4-like bacteriophages IME11, *Escherichia coli* phage IME-EC2, *Enterococcus faecium* phage IME-EFm1, and *Serratia marcescens* phage IME-SM1 have fixed termini. Only one forward and reverse HFS are located at the two ends of T3, IME-EC-16, IME-EC-17, IME-EF3, IME-EFm1, IME-SF1, IME-SF2, IME-SM1, one forward HFS at the left end of N4-like phage IME11, *S. aureus* phage IME-SA1, IME-SA2, IME-SA3, and one reverse HFS at the right end of IME-EC2, *E. faecalis* phage IME-EF4 (unique termini). N4-like phage IME11 has two right ends (multi-termini), *S. aureus* phage IME-SA1, IME-SA2, IME-SA3 have random right ends and *E. faecalis* phage IME-EF4 and IME-EC2 have random left end (Red: forward, Black: reverse, dot: preferred termini, plus sign: fixed but with unique differences in the forward and reverse termini, rhombus: unique termini at both ends).

NGS can also serve as a practical tool to find additional terminal characteristics such as terminal repeat sequences. Bacteriophage T3 had 230 bp direct terminal repeats at the two ends (Figure 8) while in N4-like phage IME11 the forward and reverse termini were separated by 401 bp direct terminal repeats (Figure 4, Figure 5). These repeat sequences were identified by mapping the forward and reverse HFSs to the genome. Further, the lengths of the repeats were calculated using the formula: repeat length = reverse termini position – forward termini position (if orientation is incorrect, genome length can be included into calculation). For example, repeat length in IME11 was: R63123– F62722 = 401 bp, for IME-EF3: G41118– R32742– F7836 = 540 bp, for IME-EC-16: R33349– (G38870– F5668) = 147 bp, in IME-EC-17: G38435– F38320– R34 = 81 bp, in IME-EC2: G41510- R38179 - F37875 = 304 bp, IME-EFm1: R42598– F42333 = 265 bp, IME-SF1: R23343– (G38842– F15658) = 159 bp, IME-SF2: R4615– F4426 = 190 bp, and in IME-SM1: R72160– F71673 = 487 bp. The long terminal redundancy region (LTR) in *S. aureus* phages IME-SA1 and IME-SA2 were about 8 kb, which was observed by mapping all sequenced reads to their assembled genome. Note that the repeat lengths of IME-AB2, IME-AB3, IME-SL1, IME08, IME09, IME-EC1, and iridovirus W150 were not calculated because of the lack of fixed termini while IME-EF4 had only the reverse terminus (Table 5, Figure 9).

Previous studies suggested that N4-like phages had a unique sequence at the left end while the right end had permutations with six groups of terminal sequences, which were caused by the asymmetric cleavage of the terminase. Such heterogeneity in the terminal sequences at the right end of N4-like phages illustrate the multi-termini of the bacteriophage genome [6]. Indeed, we found that T4-like phages also possess the multi-termini phenomenon similar to N4-like phages. Among the top 15 forward and reverse HFSs, only one HFS was present in the left end of *S. aureus* phage IME-SA1, IME-SA2, and IME-SA3, one in the right end of IME-EF4 and IME-EC2 and both right and left in the two ends of bacteriophages T3, IME-EC-16, IME-EC-17, IME-EF3, IME-EFm1, IME-SF1, IME-SF2, and IME-SM1 (T1/T2>3, unique termini, Table 5). However, N4-like phage, IME11, had one on the left (T1-F/T2-F = 4.81>3, unique termini) and two on the right (T1-R/T2-R = 1.33<3, multi-termini), which was consistent with the characteristics of N4-like phage termini (Figure 5, Table 5), and *S. aureus* phages IME-SA1, IME-SA2, and IME-SA3, which had no termini on their reverse (T1-R/T2-R = 1.38<3, no-termini). T4-like phages have obvious multi-termini in their genomes and the top 15 forward and reverse HFSs are distributed throughout the whole genome of all phages analyzed (T1/T2<3, multi- termini) (Table 5, Figure 9). Therefore, we presumed that T1/T2>3 referred to the phage with unique termini such as the the left end of IME11, IME-SA1, IME-SA2, and IME-SA3, and the right end of IME-EC2 and IME-EF4 and both ends of T3, IME-EC-16, IME-EC-17, IME-EF3, IME-EFm1, IME-SF1, IME-SF2, and IME-SM1. T1/T2<3 meant multi- termini (the right ends of IME11 and the whole genome of IME08 and IME09) or no termini (the right end of IME-SA1, IME-SA2, IME-SA3, the left end of IME-EC2, IME-EF4 and the whole genome of IME-AB2, IME-AB3, IME-SL1). Further, we predict that the termini type indicates the corresponding viral packaging mode: no termini, asymmetric termini at left and right ends indicate headful mode of package such as in iridovirus, T4-like phages, N4-like phages, *S. aureus* phages, and *A. baumannii* phages; and unique termini at both ends reflect a *cos* mode of packaging such as in T7-like phages.

When characterizing the HFSs of *S. aureus* phage IME-SA1 and IME-SA2, we observed the presence of a unique left end and random right ends in both phages (Table 6, Figure 9). Moreover, about 8000 bp long terminal redundancy region (LTR) existed in these two phage genomes (Figure S3), which was consistent with other *S. aureus* phage genomes in Genbank and previous studies [30]. In addition, IME-SA1 and IME-SA2 possessed the same forward terminus suggesting that *S. aureus* phages terminase cleave at the same site: GGAATTCTTTTACCTCTCTCACTCAGCCTA…(Table 6). We also determined that the *S. aureus* phages had the same terminal structure by aligning 500 bp sequences in the surrounding region.

**Table 6:** The top 5 forward and reverse sequences in the *S. aureus* phages, IME-SA1 and IME-SA2 genome.

Phage	Frequency	Position	Orientation	Read sequence
IME-SA1	961	64451	F	GGAATTCTTTTACCTCTCTCACTCAGCCTA…
	118	40494	R	CCTCCTTTTCTTAATTCTTATATAGTGTAT…
	101	10361	R	CCATATCTTTTGGCTCAAATTCTTTTTTCC…
	84	88098	F	CCTTCTCTTATTAATATTGATAAATCATGT…
	77	130742	R	CCACCTCCGCCACCGGAGCCTCCGTATTTC…
IME-SA2	626	134735	F	GGAATTCTTTTACCTCTCTCACTCAGCCTA…
	137	118514	R	ATATAACTTATTAGGTTCATATAAATCTTT…
	105	9117	R	AGAATTCATTATGTTTTCAGAATATGATGA…
	98	13230	R	AAATACAAGATGTATTAATTGAAAAGAACT…
	95	27321	F	GTAATAACACTATCTGAACCTATACTAATA…

## Discussion

Our findings based on next generation sequencing of bacteriophage and viral genomes revealed that HFSs are indeed the viral genome termini. Further, we used NGS data i) to determine simultaneously the complete sequence of viral genomes as well as their terminal sequences, ii) to establish a criterion to distinguish the type of termini and the viral packaging mode that exist in each bacteriophage and virus, iii) to detect additional details of terminal sequences such as terminal repeats, secondary termini and multi-termini, iv) to assess the ends of the iridovirus, W150, genome that has highly preferred sequences instead of random sequences as in T4-like phages. Using these techniques, we determined for the first time the presence of a unique left end and random right ends in *S. aureus* phages IME-SA1, IME-SA2, and IME-SA3, and the same terminase cleavage site and terminal structure in *S. aureus* phages. In addition, we also proposed a formula to calculate the terminal repeat length.

Our results on T4-like phages differed slightly from previous reports and the likely explanation for this inconsistency are explained below. The phylogenetic tree (Figure 1) constructed with large terminase subunit amino acid sequences showed the clustering of IME08, IME09 and IME-EC1 with typical T4-like phages T4, JS10, JS98, RB14, RB51 etc. indicating that these phages (IME08, 09, EC1) are T4-lke phages. Therefore, they are likely to have circular and permuted genomes as other T4-like phages. IME08, IME09 and IME-EC1 clustered with different phages representing different species within the T4-like phage genus. We thus propose that the common characteristics such as circular and partially permuted genomes with same terminase cleavage mechanism shared by IME08, IME09, and IME-EC1 could be the characteristic feature of the T4-like phage genus.

As suggested, cleavage of viral genome by terminases is a complex process. The cleavage site consists of a binding site, which is recognized by the small terminase, whereas the large terminase introduces nicks in the DNA strands at a site adjacent to the binding site. In phages like lambda, the latter occurs strictly at a specific sequence. In headful phages such as P22, SF6, and SPP1, the binding site has sequence specificity but the cleavage site is not sequence specific. In the “strictly headful” phage T4, even the binding site may not have sequence specificity [3]. A limitation of this study is that we did not analyze the binding sites, although the start sites of HFSs more likely correspond to cleavage sites on the phage genome of the large terminase. Possible explanation for this were: (i) a tag sequence was ligated to the IME09 genome before sequencing, and sequencing results clearly demonstrated that the dominant tag sequences (adaptors ligated to sequences, which had two naked ends before DNA fragmentation) were identical to the HFSs obtained from the untagged parallel experiment (Table 4). (ii) the HFSs in all T4-like phages in this study had distinct consensus sequence compared to phages with circular and completely permuted genome, such as in IME-AB2 (Figure S2). This suggested that the cleavage of T4-like genomes was not completely random and therefore we referred to this as a partially permuted genome, or at least a majority of the T4-like phage genomes are derived from partially specific (preferred) cutting at the consensus sequences. Furthermore, (iii) the conclusion that HFSs are genome termini was also supported by the experiment with adapter-tagged T3 genome and many other phage genomes (IME-SA1, IME-SA2, IME-EC2) analyzed in this study, which was consistent with the terminal sequences reported by other groups [30].

Phylogenetic tree of terminases constructed by aligning their amino acid sequences reveal clustering of large terminase into groups with similar packaging strategies and a separate cluster formed by terminases with similar enzymatic end-generating functions [8]. As demonstrated in Figure 1, the phage IME-EC2 isolated in our laboratory shared the same packaging mechanism with well-known P22-like phages, SPP1 and ES18. Our data showed that this phage genome had a definite start site (Table S4, with a few base variations) and partially permuted ending sites with a terminal redundancy of about 300 bp (about 1% of the genome), which was consistent with the headful packaging mechanism. Same terminal characteristic was also observed in *S. aureus* phages IME-SA1 and IME-SA2, which had about 8 kb terminal redundancy (Figure S3) that is concordant with previous studies on *Staphylococcus* Twort-like phages such as A5W and staph1N with long terminal repeat region (LTR) about 8 kb in length [30]. Such LTR was also observed in the well-known *Bacillus* phage, SPO1, and phylogenetic analysis indicated that *S. aureus* phages IME-SA1, IME-SA2, and IME-SA3 possess the same packaging mechanism as SPO1, which reiterates our strategy [30], [31].

Theoretically, if initiation occurred at the preferred HFS sites, then there should be islands of HFS peaks at the reverse end, each approximately correlating with the genome length of that phage. Indeed, HFS peaks in the reverse ends were observed in the headful phage, IME-EC2 (Table S4), as well as IME-SA1 and IME-SA2 (Table 6). As mentioned above, the distance between the forward HFS (start site, with one very high occurrence) and the reverse HFSs were about 300 bp in IME-EC2 (Table S4) and 8000 bp in IME-SA1 and IME-SA2 (Figure S3), which also indicated that both 300 and 8000 bp teminal redundancies existed in IME-EC2 and *S. aureus* phages, IME-SA1/IME-SA2, respectively. However, in T4-like phages it is complicated by the fact that there were many HFSs along the genome (Figure S4) in both forward and reverse directions. Therefore, it was not possible to conclude whether certain reverse HFS matched a particular forward HFS. As hypothesized, it is possible that T4-like phages presumably adopt the headful mechanism, which makes the ending termini prone to permutations and thereby result in a lack of HFSs.

All tailed phages characterized thus far show directionality in packaging. In other words, the packaging machinery starts at a specific point in the genetic map (“left” end) and terminates at a headful length distance (“right” end) [3]. According to this, there should be no HFS reads on the reverse end of the T4-like phage genome with preferred cutting sites, since termination of packaging is headful dependent. Yet, we observed HFS reads on the reverse end indicating that T4-like phages do not have obvious directionality in packaging. Some likely interpretations for this are discussed here. First, since our raw data collected was large covering various phage genera, and we performed subsequent confirmation experiments with tagged T3 genome with known termini, as well as tagged T4-like IME09 genome, we believe that T4-like phages (at least IME08, IME09 and IME-EC1) should have forward and reverse HFSs at both termini of the genome. Second, all data from these three phages support this hypothesis. In our previous study on IME08, we discovered that almost all the HFSs started with a G or A base. Interestingly, all the highest frequency sequences started with G [13], [30]. Further analysis also showed that cleavage was at the consensus sequences, which led us to conclude that these HFSs may represent the genome termini. Therefore, in this study we designed a tag sequence to ligate to the IME09 genome and compared its sequence with the non-tagged IME09 genome sequence. The results revealed that the tagged sequences are indeed the HFSs. Third, for different T4-like phages, the consensus sequences are different (Figure S2) suggesting that physical or chemical shearing could not have played a role. This hypothesis was verified by sequencing a known terminal phage T3 as discussed above. Fourth, these HFSs are not likely to be artifacts because we found them only in the sequences of phage genomes but not in any other genome such as several bacterial genomes sequenced using the same protocol (data not shown). Fifth, we believe these are not random results, since HFSs were not identified in some phages like IME-AB2 (Table 5, Figure S2), even with genome smaller than IME08, IME09, and IME-EC1.

When characterizing the HFSs of *S. aureus* phage IME-SA1, IME-SA2 and IME-SA3, we observed that the forward terminal sequence occurred more frequently than the reverse terminal sequence. Among the top 15 forward and reverse HFSs in IME-SA1 and IME-SA2 and IME-SA3 (Figure 9), the forward HFSs occurred about 5 times more than the reverse suggesting that the 5′ terminus was less permuted than the 3′ terminus. The same phenomenon was observed previously in T4-like bacteriophage IME08 and N4-like phages where the 5′ terminus was more unique than 3′ terminus [6]. This uniqueness between the forward and reverse sequences indicated that the genomic DNA packaging was asymmetric. We propose two probable reasons for this i) some protection mechanism may exist in the ends of the bacteriophage genome. Diverse protection mechanisms are employed to protect the genome ends from cleavage by terminase. For example, bacteriophage N4 has hairpin priming at the 3′ terminus of the genome, which makes it difficult for the terminase to cut the right end, but the left end without such protection is easily cleaved [6]. Other types of terminal protection include proteins attached to genomic ends such as in phage Φ29 and adenoviruses, and in phage Mu host genomic DNA is adhered to the ends of the phage genome [32], [33], [34]. ii) The recombination-dependent DNA replication could be asymmetric such as in T4-like bacteriophage, which makes one of the ends dominant during packaging [35].

The core and the most important element of the viral genome are the termini, which are involved in almost the whole life cycle starting from host infection to packaging of virions and their release from the host cell by lysis. The termini play important roles during viral DNA replication [32], packaging, initiation [3], termination and regulation of transcription [31], [36], [37], [38], metabolism of host cell [39], and serve as binding domains for specific proteins such as RNA polymerases [31], [37].

Structure of the bacteriophage genome termini is closely associated with their DNA replication, which proceeds from 5′ to 3′ end. Some special mechanisms must exist at the termini of their genomes to guarantee cycles of regular DNA replication. For example i) the genome of bacteriophage N4 has about 500 bp unique direct terminal repeats, and non-complementary extensions in both 3′ and 5′ ends [6]. On the 3′ ends, there are some inverted repeats, and Y-shaped molecules exist in the various extensions, which initiate N4 DNA synthesis at the ends of the N4 genome, preferentially at the right end [6]. ii) Phage T7 has direct terminal repeats [3], [7], [8], which are used to form concatemers, the intermediary form in DNA replication. iii) In bacteriophage λ, the cohesive ends can be covalently closed into a circular form, which initiates DNA replication [5]. iv) Adenovirus and PhageΦ29 have covalently bound proteins on each end of the genome (provide a C-OH), which serve as primers to initiate replication [8], [32], [33].

In addition, the terminal redundancy of some phages plays important roles in redirecting the metabolism of the host to phage production during a “host-takeover” process [31], [39]. In *Bacillus subtilis* bacteriophage SPO1 terminal redundancy (TR), there is a cluster of early genes (24 genes) named 11.5 kb “host-takeover module” [39]. Since a majority of early transcription takes place within the 11.5 kb terminal redundancy, the 24 early genes encoding proteins are involved in the shutdown of host macromolecular DNA, RNA, and protein biosynthesis in the early infection of the phage SPO1 [36]. There are also additional functions for the TR’s early gene, such as the shut off rate limiting function of bacteriophage SPO1 gene 39 and the shut off accelerating role of bacteriophage SPO1 gene 40 [37], the regulation of gene expression by bacteriophage SPO1 genes 44, 50, and 51 [38], and other functions such as transcription-termination and RNase III cleavage, zinc finger, and RNA polymerase-binding domains [39].

With the importance of genomic termini in viruses and bacteriophages being increasingly recognized, research on virology, genomics, phage biology, and phage therapy should target genome termini in future studies. Traditional methods to study viruses and bacteriophages include construction of recombinant plasmids, expression of recombinase, labeling of terminal biotin [6], [7], [9], site-directed mutagenesis [37], and molecular hybridizations such as Northern blots [36]. Using these methods to prove the theory of terminase cleavage will require significant time and personnel, and easy to lose some important characteristics of the genome. However, recent advances in high-throughput sequencing have made its application easier, cheaper, more convenient and more efficient allowing it to evolve into a powerful tool for termini study. For this approach, we used simple strategies such as genomic DNA extraction, NGS, mapping of the reads to the assembled genome and HFSs occurrence calculation. When conducting this study, we noticed that critical factors for NGS are integrity of the genome (used for adaptor tagging) without any degradation, and the sample processing without repeated freeze/thaw. In addition, since the termini of the bacteriophage genome were polished blunt in our experiment, it is possible that we lost the details in protrusions.

In conclusion, we report for the first time that the high frequency sequences identified in the next generation sequencing are indeed terminal sequences of the bacteriophage genomes. These terminal sequences can be obtained either with or without tagging the adaptor to phage genome. Interestingly, our analysis indicated that the ends of the T4-like phage genome were cleaved in a sequence preferred manner instead of random cleavage, which is in disagreement with previous assertions. Moreover, we observed unique differences in the forward and reverse termini of the genome indicating asymmetry during packaging partly explaining the existence of multi-termini in the bacteriophage genome. We also identified the presence of specific terminal sequences in N4-like bacteriophage using the NGS method. With this approach we were able to simultaneously detect details in the genome terminal sequences such as terminal repeats, secondary termini, multi-termini, as well as obtain the complete sequence of the bacteriophage genome thus simplifying the analysis. Theoretically, this application could be further extended to simultaneously analyze the complete genome and the terminal sequences of plant and animal viruses, although the genome sequences could be larger and complicated. This study shows a fast and efficient method for research on viral replication, packaging, function of terminase, transcription regulation, and metabolism of host cell.

## Supporting Information

Figure S1 **Percentage of first bases in read sequences with/without adaptor tag.**
(PDF)

Figure S2 **Consensus sequences comparison of T4-like phages and** ***Acinetobacter baumannii*** **phage, IME-AB2.**
(PDF)

Figure S3 **Mapping of** ***S. aureus*** **phage, IME-SA1 and IME-SA2.**
(PDF)

Figure S4 **Distribution of top 100 forward and reverse HFSs on the IME09 genome.**
(PDF)

Table S1 **Differences in the T3 phage genome sequenced by high throughput sequencing (Illumina) and previously published sequence in GenBank (NC_003298, sequenced by ABI 377 DNA analyzer).**
(DOC)

Table S2 **The frequency percentage of IME09 reads with/without adaptor.**
(PDF)

Table S3 **Comparison between HFSs and consensus sequences in the IME09 genome.**
(PDF)

Table S4 **The top 7 forward and reverse sequences in the phage IME-EC2 genome.**
(PDF)
