# Supplementary material for: Scrutinizing Virus Genome Termini by High-Throughput Sequencing
Source: PLoS One. 2014 Jan 20;9(1):e85806. doi: 10.1371/journal.pone.0085806 (PMC3896407; doi:10.1371/journal.pone.0085806)
Supplement: Figure S1 — Percentage of first bases in read sequences with/without adaptor tag. (PDF) [file pone.0085806.s001.pdf]

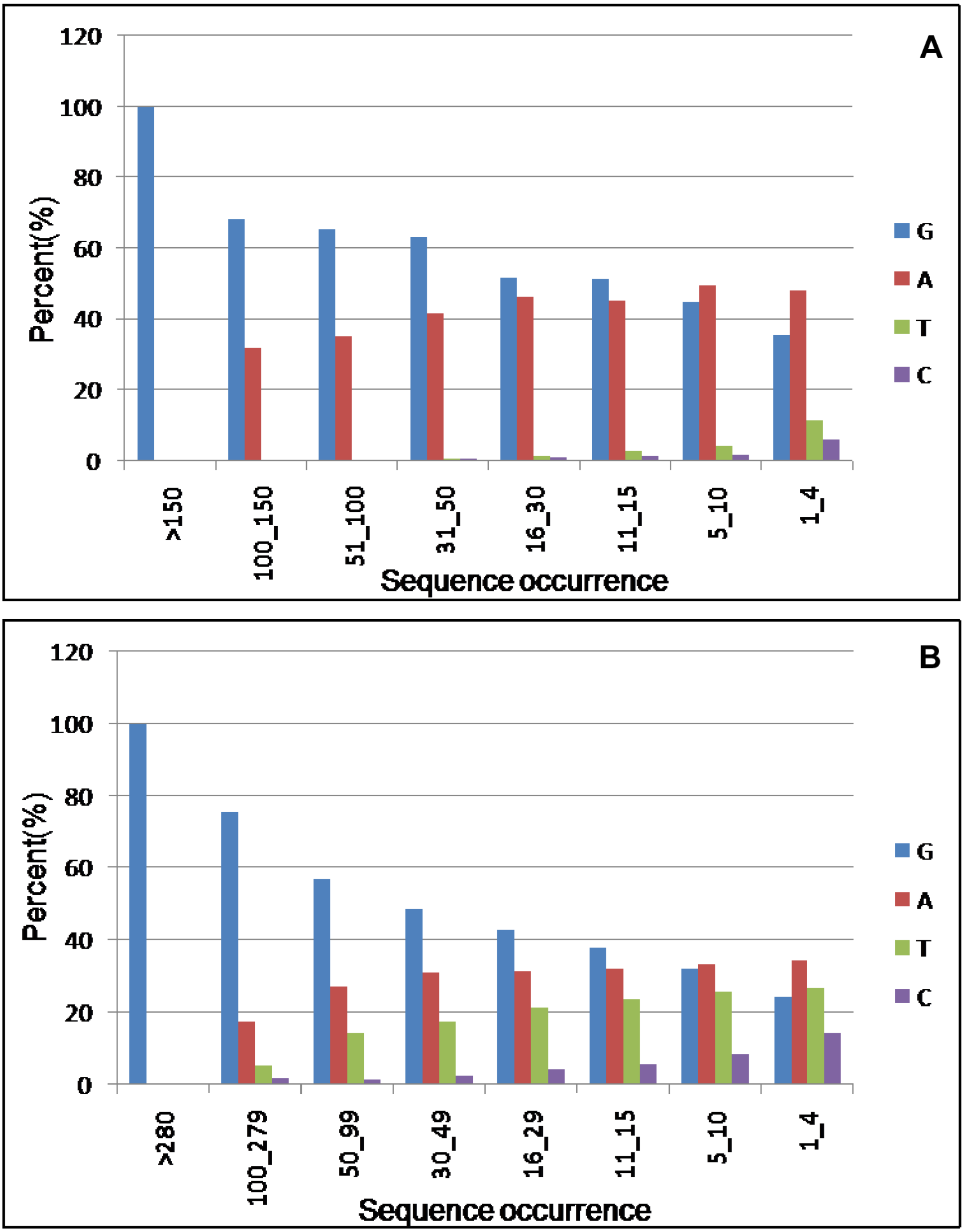

Figure S1. Percentage of first bases in read sequences with/without adaptor tagged. (A) read sequences with 1-4 occurrences has A, T, C, G constitution as the genome base composition (more evident in B), however, A and G comprised more than 90% of the first base for the sequences occurred more than 11 times, and there was never a T or C base for sequences occurred more than 100 times. **(B)** read sequences with 1-4 occurrences has A, T, C, G constitution as the genome base composition. A and G comprised more than 90% of the first base for the sequences occurred more than 100 times, and there was never a T or C base for sequences occurred more than 280 times.
