# Supplementary material for: Scrutinizing Virus Genome Termini by High-Throughput Sequencing
Source: PLoS One. 2014 Jan 20;9(1):e85806. doi: 10.1371/journal.pone.0085806 (PMC3896407; doi:10.1371/journal.pone.0085806)
Supplement: Figure S2 — Consensus sequences comparison of T4-like phages and Acinetobacter baumannii phage, IME-AB2. (PDF) [file pone.0085806.s002.pdf]

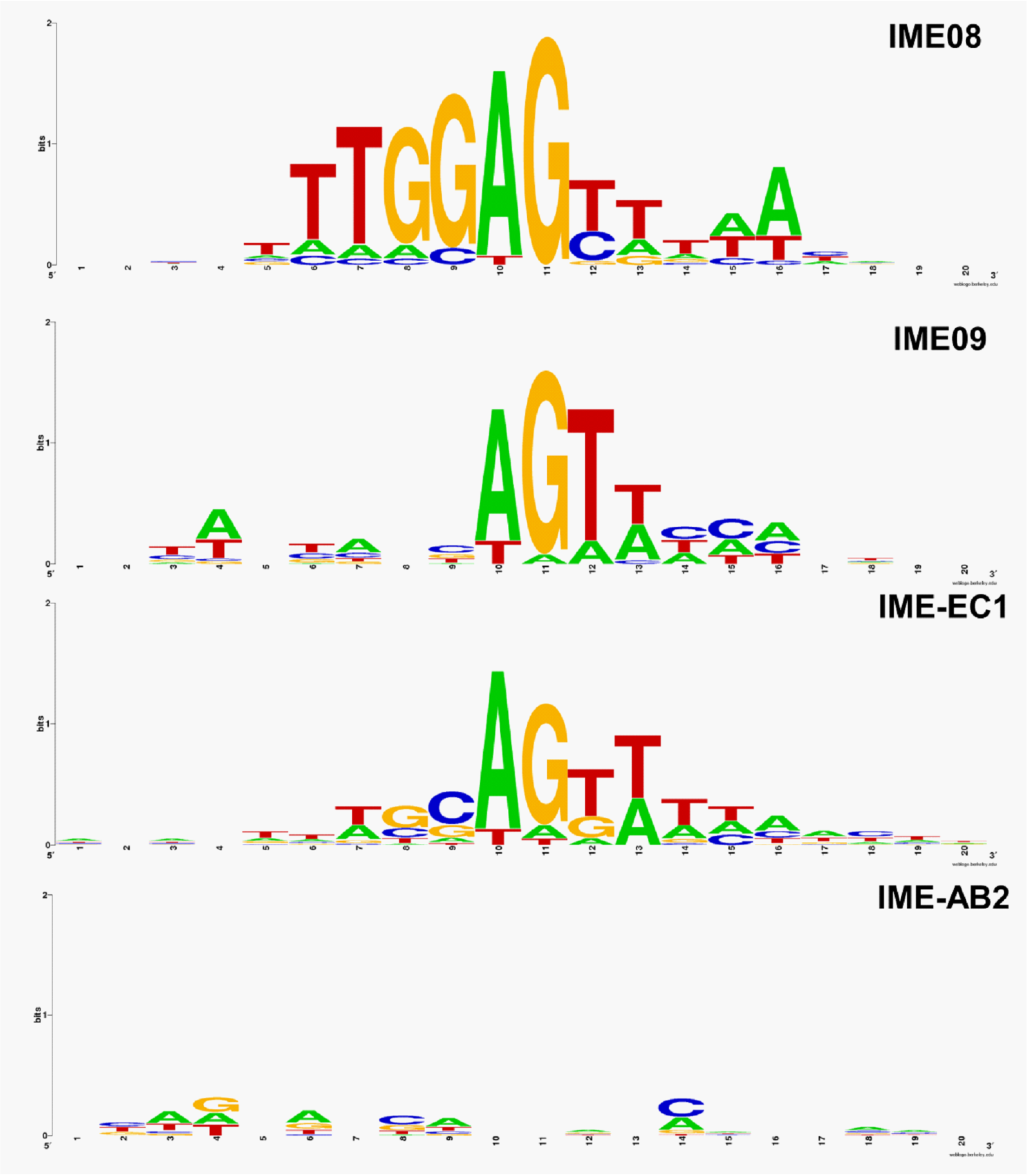

Figure S2. the consensus sequences comparison of T4-like phages and *Acinetobacter baumannii* phage IME-AB2. T4-like phage IME08, IME09, IME-EC1 were assumed aspartially permuted genome and *Acinetobacter baumannii* phage IME-AB2 was assumed as completely permuted circular genome which without any evident HFSs.
