# Supplementary material for: Scrutinizing Virus Genome Termini by High-Throughput Sequencing
Source: PLoS One. 2014 Jan 20;9(1):e85806. doi: 10.1371/journal.pone.0085806 (PMC3896407; doi:10.1371/journal.pone.0085806)
Supplement: Figure S3 — Mapping of S. aureus phage, IME-SA1 and IME-SA2. (PDF) [file pone.0085806.s003.pdf]

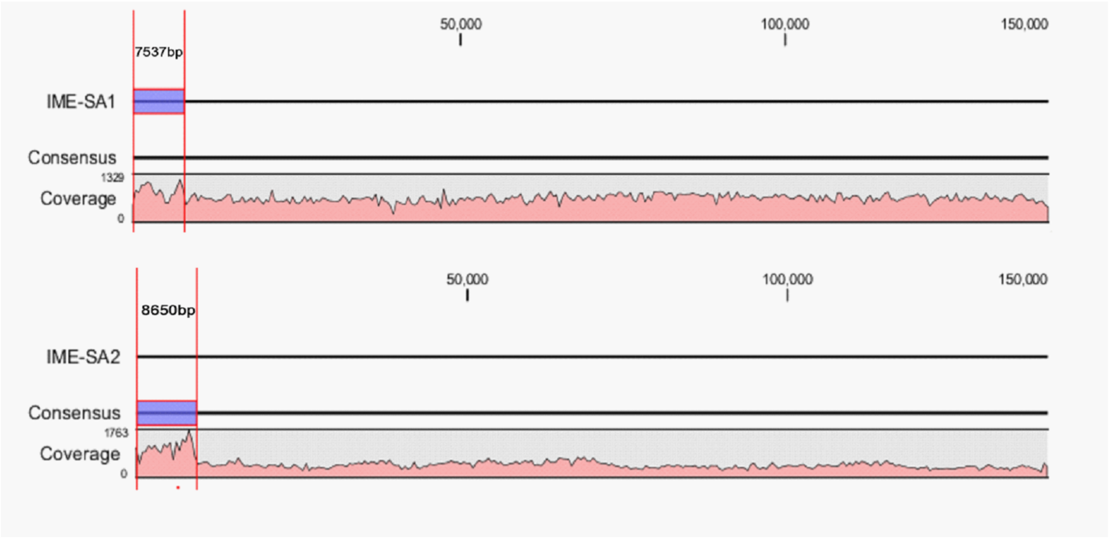

Figure S3. The mapping of *S. aureus* phage IME-SA1 and IME-SA2. *S. aureus* phage genome has about 8000bp long terminal repeats at the ends of their genome.
