# Supplementary figures and images for: Scrutinizing Virus Genome Termini by High-Throughput Sequencing
Source: PLoS One. 2014 Jan 20;9(1):e85806. doi: 10.1371/journal.pone.0085806 (PMC3896407; doi:10.1371/journal.pone.0085806)

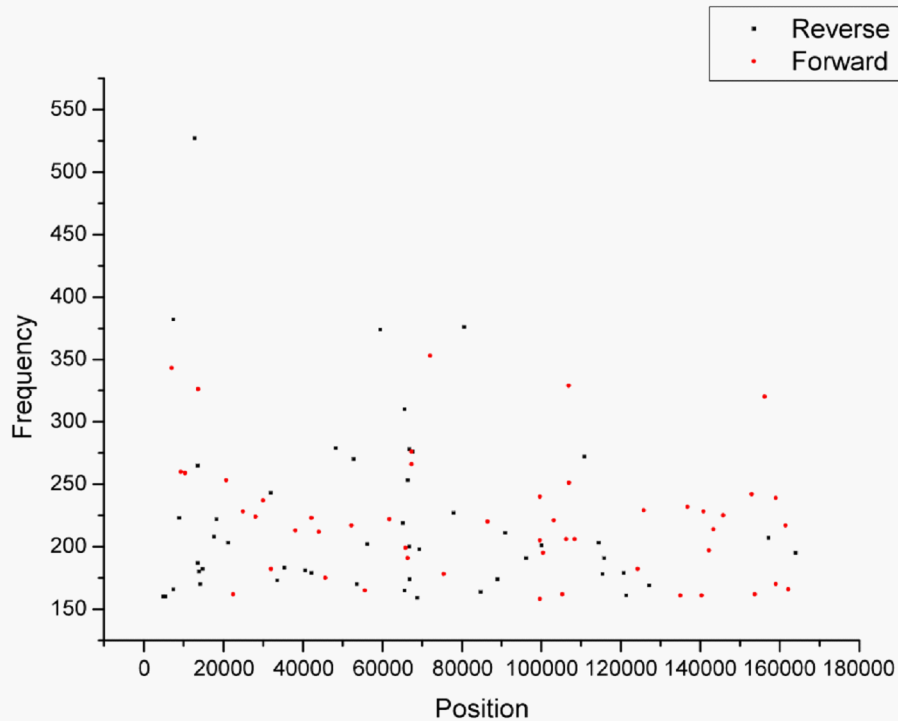

**Figure S4. Distribution of top 100 forward and reverse HFSs on the IME09 genome**

Supplement: Figure S4 — Distribution of top 100 forward and reverse HFSs on the IME09 genome. (PDF) [file pone.0085806.s004.pdf]
