# Supplementary material for: Scrutinizing Virus Genome Termini by High-Throughput Sequencing
Source: PLoS One. 2014 Jan 20;9(1):e85806. doi: 10.1371/journal.pone.0085806 (PMC3896407; doi:10.1371/journal.pone.0085806)
Supplement: Table S1 — Differences in the T3 phage genome sequenced by high throughput sequencing (Illumina) and previously published sequence in GenBank (NC_003298, sequenced by ABI 377 DNA analyzer). (DOC) [file pone.0085806.s005.doc]

Supplement 1. Differences in the T3 phage genome sequenced by high throughput sequencing (Illumina) and previously published sequence in GenBank (NC_003298, sequenced by ABI 377 DNA analyzer)

| T3 sequence position | Gene position | Base variation | Amino acid variation | Mutation type |
| --- | --- | --- | --- | --- |
| 33 | noncoding region | C-->/ | Non-coding | Single base deletion |
| 718 | noncoding region | T-->C | Non-coding | transition |
| 2263 | gene 0.7 | C-->T | No change | synonymous mutations |
| 9603 | gene 3 | T-->TGC | Local frameshift | double bases insertion |
| 9622 | gene 3 | C-->CG | Frameshift rescue | single base insertion |
| 9969-9973 | gene 3 | TGGCG-->GTGGC | AGV-->AWL | five bases replacement |
| 13564 | gene 5 | C-->A | D-->E | missense mutation |
| 19676 | gene 8 | C-->A | No change | synonymous mutations |
| 19677 | gene 8 | A-->C | K-->Q | missense mutation |
| 20725 | gene 9 | T-->C | No change | synonymous mutations |
| 22151 | gene 10b | T-->C | S-->P | missense mutation |
| 22169 | gene 10b | T-->C | No change | synonymous mutations |
| 23103 | gene 12 | A-->G | T-->A | missense mutation |
| 23154 | gene 12 | A-->C | I-->L | missense mutation |
| 24243 | gene 12 | G-->A | D-->N | missense mutation |
| 24657 | gene 12 | A-->G | R-->G | missense mutation |
| 25495 | noncoding region | A-->G | Non-coding | transition |
| 28858 | gene 16 | A-->C | E-->A | missense mutation |
| 38010 | noncoding region | C-->/ | Non-coding | single base deletion |
