# Supplementary material for: Scrutinizing Virus Genome Termini by High-Throughput Sequencing
Source: PLoS One. 2014 Jan 20;9(1):e85806. doi: 10.1371/journal.pone.0085806 (PMC3896407; doi:10.1371/journal.pone.0085806)
Supplement: Table S2 — The frequency percentage of IME09 reads with/without adaptor. (PDF) [file pone.0085806.s006.pdf]

Table S2. The frequency percentage of IME09 reads with/without adaptor

frequency	reads/with adaptor	percentage	reads/without adaptor	percentage
1	221234	61.40%	69938	69.30%
2	55807	15.50%	12096	12%
3	29052	8%	5349	5.30%
4	16233	4.50%	2985	2.96%
5	9379	2.60%	2025	2%
6	5842	1.60%	1445	1.43%
7	3988	1.10%	1095	1.08%
8	2859	0.80%	789	0.78%
9	2053	0.60%	679	0.67%
10_99	13280	3.78%	4422	4.38%
above 100	312	0.09%	60	0.06%
