# Supplementary material for: Scrutinizing Virus Genome Termini by High-Throughput Sequencing
Source: PLoS One. 2014 Jan 20;9(1):e85806. doi: 10.1371/journal.pone.0085806 (PMC3896407; doi:10.1371/journal.pone.0085806)
Supplement: Table S3 — Comparison between HFSs and consensus sequences in the IME09 genome. (PDF) [file pone.0085806.s007.pdf]

Table S3 The comparison between HFSs and consensus sequences of IME09 genome.

Sequences	Calculated results
HFSs (calculated from raw reads)	360049 (s)
Genome length	166499 (bp)
Consensus sequences (extracted by degenerate motif finder)	25227 (forward: 12556, reverse: 12671)
Matched	19215(forward: 11031, reverse: 8184)
