# Supplementary material for: Scrutinizing Virus Genome Termini by High-Throughput Sequencing
Source: PLoS One. 2014 Jan 20;9(1):e85806. doi: 10.1371/journal.pone.0085806 (PMC3896407; doi:10.1371/journal.pone.0085806)
Supplement: Table S4 — The top 7 forward and reverse sequences in the phage IME-EC2 genome. (PDF) [file pone.0085806.s008.pdf]

Table S4. The top 7 forward and reverse sequences in the phage IME-EC2 genome

Orientation	Frequency	Position[1]	Read sequence
	695	**38179**	AAATCAGGCGTCGCGGATATCTCGCGTATG
Reverse	99	38177	ATCAGGCGTCGCGGATATCTCGCGTATGTA
	79	38180	GAAATCAGGCGTCGCGGATATCTCGCGTAT
	77	38181	AGAAATCAGGCGTCGCGGATATCTCGCGTA
	66	**37880**	ATCAGCTTGATCTCCTCGGTGGTCATTGGC
Forward	63	37873	CTCCGCCATCAGCTTGATCTCCTCGGTGGT
	62	37879	CATCAGCTTGATCTCCTCGGTGGTCATTGG

[1]Reverse has definite HFSs with several base variation, while the HFSs on forward were not evident as reverse, which was 10 times with the forward, that indicating the reverse fixed while the forward was sligthly random. The direat repeat length = 38179 (R) – 37880 (F) =299bp
